# Preliminary Results on the Effects of American Elderberry Juice for Improving Cognition and Inflammatory Markers in Mild Cognitive Impairment

**DOI:** 10.1002/alz.083789

**Published:** 2025-01-09

**Authors:** Madison Musich, Ashley F. Curtis, Bradley J. Ferguson, Andrew L. Thomas, C. Michael Greenlief, Joel I. Shenker, David Q. Beversdorf

**Affiliations:** ^1^ University of Missouri, Columbia, MO USA; ^2^ University of South Florida, Tampa Bay, FL USA

## Abstract

**Background:**

Despite data showing nutritional interventions high in antioxidant/anti‐inflammatory properties (anthocyanin‐rich foods such as blueberries/elderberries) may decrease risk of memory loss and cognitive decline, evidence for such effects in mild cognitive impairment (MCI) patients is limited. Therefore, we examined preliminary effects of American elderberry (*Sambucus nigra canadensis*) juice on cognition and inflammatory markers in MCI patients in a double‐blind placebo‐controlled trial.

**Method:**

MCI patients (*N* =  24, *M*_age_ =  76.33±6.95) received elderberry (*n* =  11) or placebo (*n* =  13) juice (5mL orally 3‐times‐a‐day) for 6 months. At baseline, 3‐month, and 6‐month follow‐ups, patients completed tasks assessing global cognition, verbal memory, language, cognitive flexibility, and visuospatial memory. A subsample (*N* =  12, 7 elderberry/5 placebo) provided blood samples at these timepoints to measure plasma inflammatory markers. Multilevel models examined effects of condition (elderberry/placebo), time (baseline/3‐months/6‐months) and condition by time interactions on cognition/inflammation outcomes. Interactions were evaluated via Kenward‐Roger pairwise tests.

**Result:**

For cognition, the condition by time interaction trended towards significance for Visuospatial Problem Solving (VPS)‐mean latency correct (*p* = .08). Elderberry (not placebo) trended (*p* = .09) towards a decrease (faster performance in seconds) from baseline (*M* =  43.31, *SD* =  34.41) to 6‐months (*M* =  23.20, *SD* =  14.45). For inflammation, the condition by time interaction was significant for L‐lactate dehydrogenase A chain (LDHA; *p* = .03) and complement factor D (CFD; *p* = .03). In the placebo condition, LDHA significantly (*p* = .004) decreased from baseline (*M* =  4.78, *SD* =  1.41) to 6‐months (*M* =  2.07, *SD* =  0.89), while for elderberry LDHA remained stable (*p*s>.05). CFD significantly (*p* = .04) increased from 3‐months (*M* =  4.52, *SD* =  1.37) to 6‐months (*M* =  7.52, *SD* =  1.79) for placebo, while for elderberry, CFD trended (*p* = .051) towards decrease from baseline (*M* =  6.80, *SD* =  2.35) to 6‐months (*M* =  4.34, *SD* =  2.95). CFD was significantly higher for placebo vs. elderberry at 6‐months (*p* = .02). Exploratory correlation analyses (*n* =  12) showed a significant negative association (*r* = ‐0.93, *p* = .02) between change scores (6‐months–baseline) for LDHA and VPS‐mean latency correct in the elderberry condition.

**Conclusion:**

Elderberry (not placebo) juice consumption in MCI patients may provide some benefit to cognitive flexibility. Preliminary analyses reveal this benefit may be related to reduction of low‐grade inflammation. These promising findings provide support for larger, more definitive prospective studies with longer follow‐ups to better understand mechanisms of action and the clinical utility of elderberries for mitigating cognitive decline.

